# Fachkliniken – Definition: Vorschlag zur Diskussion aus der Ad-hoc-Kommission Versorgungsstrukturen der Arbeitsgemeinschaft der Wissenschaftlichen Medizinischen Fachgesellschaften (AWMF) zur Reform der Krankenhausversorgung

**DOI:** 10.3205/000325

**Published:** 2023-07-05

**Authors:** Winfried J. Randerath, Martin Kriegmair, Andreas Markewitz, Burkhard Rodeck, Thomas Schmitz-Rixen, Anton Scharl, Monika Nothacker

**Affiliations:** 1 Deutsche Gesellschaft für Pneumologie und Beatmungsmedizin (DGP e.V.), Berlin, Deutschland; 2 Institut für Pneumologie an der Universität zu Köln, Solingen, Deutschland; 3 Deutsche Gesellschaft für Urologie (DGU e.V.), Berlin, Deutschland; 4 Urologische Klinik München-Planegg, Planegg, Deutschland; 5 Deutsche Interdisziplinäre Vereinigung für Intensiv- und Notfallmedizin (DIVI e.V.), Berlin, Deutschland; 6 Deutsche Gesellschaft für Thorax-, Herz- und Gefäßchirurgie (DGTHG e.V.), Berlin, Deutschland; 7 Deutsche Gesellschaft für Kinder- und Jugendmedizin (DGKJ e.V.), Berlin, Deutschland; 8 Deutsche Gesellschaft für Chirurgie (DGCH e.V), Berlin, Deutschland; 9 Deutsche Gesellschaft für Gynäkologie und Geburtshilfe (DGGG e.V.), Berlin, Deutschland; 10 Klinik Bad Trissl, Oberaudorf, Deutschland; 11 Arbeitsgemeinschaft der Wissenschaftlichen Medizinischen Fachgesellschaften (AWMF e.V.), Berlin, Deutschland; 12 Institut für Medizinisches Wissensmanagement (IMWi), Marburg, Deutschland

**Keywords:** specialist clinics, international classification of diseases (ICD), German procedure classification (OPS), structure element, patient care

## Abstract

Specialist clinics play an important role in inpatient care in the German healthcare system. They are characterized by a high degree of specialization and expertise and are described in numerous medical disciplines. However, a generally accepted legal definition of specialist clinics has so far been lacking. The ad hoc committee of the Association of the Scientific Medical Societies in Germany (AWMF) for the reform of hospital care in Germany, therefore, presents proposals for a definition, a description of quality characteristics, and an allocation to care levels. In the opinion of the committee, three aspects are necessary for the definition of specialist clinics: a high total number of patients treated, a high proportion of the disease entities attributable to the specialist area according to diagnosis and services, and structural characteristics. The definition proposed here is intended to help the political decision-makers in the federal and state governments to standardize according to medical-scientific criteria.

## Abstract (en)

Fachkliniken spielen in der stationären Versorgung im deutschen Gesundheitswesen eine wesentliche Rolle. Sie zeichnen sich durch eine hohe Spezialisierung und Expertise aus und werden in zahlreichen medizinischen Disziplinen beschrieben. Allerdings fehlt bisher eine allgemein akzeptierte Legaldefinition von Fachkliniken. Die Ad-hoc-Kommission der Arbeitsgemeinschaft der Wissenschaftlichen Medizinischen Fachgesellschaften (AWMF) zur Reform der Krankenhausversorgung in Deutschland legt daher Vorschläge für eine Definition, die Beschreibung von Qualitätsmerkmalen und die Zuordnung zu Versorgungsebenen vor. Drei Aspekte sind zur Definition von Fachkliniken nach Ansicht der Kommission notwendig: eine hohe Gesamtzahl von behandelten Patientinnen und Patienten, ein hoher Anteil der auf das Fachgebiet entfallenden Krankheitsentitäten nach Diagnose und Leistungen sowie Strukturmerkmale. Die hier vorgeschlagene Definition soll den politischen Entscheidungsträgern in Bund und Ländern helfen, eine Vereinheitlichung nach medizinisch-wissenschaftlichen Kriterien festzulegen.

## Hintergrund

Die vom Bundesministerium für Gesundheit (BMG) eingesetzte „Regierungskommission für eine moderne und bedarfsgerechte Krankenhausversorgung“ erkennt in ihrer 3. Empfehlung die Rolle der Fachkliniken für die stationäre Versorgung an und ordnet sie den Krankenhaus-Leveln II oder III zu. Sie empfiehlt jedoch auch, dass Fachkliniken baulich und strukturell in allgemeine Level II- oder III-Kliniken integriert werden sollen [1]. Dieser regelhaften Integration ist von verschiedener Seite widersprochen worden. Vielmehr ist eine spezifische, ergebnisoffene Entscheidung auf Ebene der Länder für die einzelnen Kliniken zu treffen, inwieweit eine Integration eine qualitative Verbesserung der Versorgung in einer Region erreichen kann und wirtschaftlich sinnvoll ist oder ob eine effiziente und qualitativ hochwertige Institution als Fachklinik erhalten werden sollte. Nach jüngsten Informationen plant das BMG eine besondere Berücksichtigung der Fachkliniken (Level F). Die durch die Ad-hoc-Kommission der Arbeitsgemeinschaft der Wissenschaftlichen Medizinischen Fachgesellschaften (AWMF) erarbeiteten Kriterien sollen den verantwortlichen Gremien eine Entscheidungsgrundlage bieten. 

Es wurden folgende Fragen beantwortet:

1. Wie können Fachkliniken definiert werden?
2. Welche Qualitätsmerkmale können für Fachkliniken beschrieben werden?
3. Können Fachkliniken Leveln zugeordnet werden?

Alle Aussagen beziehen sich auf somatische Kliniken.

## 1 Wie können Fachkliniken definiert werden?

### 1.1 Vorhandene Kriterien aus Bund und Ländern 

#### 1.1.1 Bund-Länder-Arbeitsgruppe für die Krankenhausreform

Die Bund-Länder-Arbeitsgruppe für die Krankenhausreform hat in ihrer Sitzung vom Februar 2023 festgehalten, dass es keine Legaldefinition des Begriffes „Fachklinik“ im Bund oder den Ländern gibt. Sie gibt Hinweise: Demnach sind Fachkliniken Krankenhäuser, die sich auf die Behandlung einer bestimmten Erkrankung oder Krankheitsgruppe spezialisiert haben, in relevantem Umfang zur Behandlung in ihrem Spezialisierungsbereich beitragen und damit über umfassende Erfahrung sowie i.d.R. eine überdurchschnittliche Behandlungsqualität verfügen [2].

#### 1.1.2 Landeskrankenhausplan NRW

Der in Umsetzung befindliche Landeskrankenhausplan NRW (LKHP NRW) beschreibt ebenfalls wie folgt Kriterien für Fachkliniken und ihre Abgrenzung von Allgemeinkrankenhäusern:

- Plankrankenhäuser
- Spezialisierung auf Leistungsbereiche/-gruppen
- Schwerpunktmäßige Abdeckung eines speziellen medizinischen Gebietes
- Kompetente Leitung
- Hochdifferenzierte Behandlung schwerer und schwerster Krankheitsbilder
- Kooperation mit Allgemeinkrankenhäusern

Abgrenzung von Allgemeinkrankenhäusern (LKHP NRW):

- Besondere Leistungsfähigkeit aufgrund der fachspezifischen Versorgung
- Hohe Fallzahl im Gebiet
- Fachspezifisch hochwertigere Ressourcen

Mindestvoraussetzungen sind anhand von Geräten, fachärztlichen Stellenanforderungen sowie Struktur- und Prozesskriterien zu beschreiben. Im Gegensatz zu Allgemeinen Krankenhäusern (AKH) können Fachkliniken Kooperationen an die Stelle weiterer Leistungsgruppen (LG) stellen. Außerdem müssen sie nach dem LKHP NRW in der Grundversorgung nicht die Kombination der LG „Allgemeine Innere Medizin“, „Allgemeine Chirurgie“ und „Intensivmedizin“ vorhalten. Im Falle von konkurrierenden LG an AKH sollen die Fachkliniken nur bei Sicherstellung der Qualitätskriterien, bei besonderer Leistungsfähigkeit im Vergleich zum AKH, bei Sicherstellung der wirtschaftlichen Tragfähigkeit und sinnvoller Einbindung in die regionale Versorgung den Vorrang bei Auswahlentscheidungen erhalten [3].

#### 1.1.3 Bayerische Landeskrankenhausplan

Der Bayerische Landeskrankenhausplan definiert Fachkrankenhäuser als nach Art der Erkrankung abgegrenzte Einrichtungen, in denen überwiegend in einer Fachdisziplin durch Gebietsärzte bestimmte Krankheiten, Leiden oder Körperschäden festgestellt, geheilt oder gelindert werden oder in denen Geburtshilfe geleistet wird [4].

### 1.2 Vorschläge der Ad-hoc-Kommission Versorgungsstrukturen der AWMF zur Definition von Fachkliniken für die Krankenhausreform

#### 1.2.1 Prinzip

Die Ad-hoc-Kommission der AWMF schlägt eine qualitative und quantitative Definition vor, die insbesondere folgende quantitativen Aspekte berücksichtigt:

1. Eine Mindestanzahl behandelter Patientinnen und Patienten, verglichen mit dem Versorgungsvolumen im spezifischen Bereich. Dies ist Voraussetzung für eine Relevanz für die Versorgung der Bevölkerung (siehe dazu Versorgungsvolumen).
2. Einen überdurchschnittlich hohen Teil der für das Fachgebiet relevanten International-Classification-of-Diseases(ICD)- oder Operationen- und Prozedurenschlüssel(OPS)-Ziffern: Dies beschreibt die umfassende Darstellung des Fachgebietes in der Breite (siehe dazu Fachklinik A).
3. Alternativ zur umfassenden Breite die hohe Spezialisierung auf einzelne, wenige Leistungen (siehe dazu Fachklinik S)
4. Die Definition von Strukturelementen: Diese werden durch die Beschreibung von Leistungsgruppen oder die Voraussetzungen für operative Leistungen definiert (siehe dazu Zuordnung zu Leveln).

#### 1.2.2 Definition von Fachkliniken – Vorschlag Ad-hoc-Kommission

- Unter Fachkliniken im Sinne der Krankenhausreform sind eigenständige Plankrankenhäuser der Akutversorgung zu verstehen, d.h. keine Einrichtungen der Rehabilitation [5]. Es handelt sich auch nicht um Fachabteilungen in allgemeinen Krankenhäusern.
- Fachkliniken decken ein spezielles medizinisches Gebiet ab oder sind auf spezielle Krankheitsbilder oder Leistungen fokussiert. Dabei sollte die von ihnen betreute Anzahl von Patientinnen und Patienten im oberen Bereich des Versorgungsvolumens ihres Gebietes oder ihres speziellen Krankheitsbildes oder der angebotenen speziellen Leistungen liegen (z.B. oberhalb der 75. Perzentile). Diese Zahl ist nur als Beispiel gesetzt und ist anhand von Versorgungszahlen zu prüfen und anzupassen (eventuell auch regional). Als Vergleichsparameter gelten die in den Leistungsgruppen angegebenen ICD-/OPS-Ziffern.
- Fachkliniken behandeln (auch) schwere, komplexe oder chronische Krankheitsbilder des Fachgebietes und bieten dabei spezialisierte Interventionen und Behandlungsverfahren an, jedoch nicht notwendigerweise mit intensivmedizinischer Versorgung.
- Fachkliniken sind auch, jedoch nicht ausschließlich, Anlaufstellen für Patientinnen und Patienten des Fachgebietes, die in einem Allgemeinkrankenhaus nicht hinreichend versorgt werden können. Dies beinhaltet die grundsätzliche Verpflichtung zur Übernahme entsprechender Patientinnen und Patienten aus anderen Häusern und die Beratung von Ärztinnen und Ärzten anderer Häuser, auch mittels telemedizinischer Angebote.
- Fachkliniken pflegen einen strukturierten Austausch mit Allgemeinkrankenhäusern.

Innerhalb der Fachkliniken gibt es Unterschiede in der Breite des Behandlungsspektrums. Daher wird die folgende Unterscheidung als sinnvoll erachtet:

- **Fachkliniken A (Allgemein)**, die ein Fachgebiet in der Breite (Gesamtspektrum) abbilden und (mindestens) fach- oder diagnosespezifische Mindestmengen (wie oben angegeben) erfüllen. Diese Fachkliniken betreuen Patientinnen und Patienten mit mindestens (z.B.) 60% des ICD-/OPS-Spektrums des Fachgebietes, inklusive seltener und komplexer Erkrankungen.
- **Fachkliniken S (Spezial)**, die sich auf die Diagnostik und Therapie einzelner Erkrankungen oder die Durchführung einzelner Behandlungen festgelegt haben.

**Hinweis:** Einige Kliniken nehmen eine Sonderrolle ein, wie Berufsgenossenschaftliche Kliniken und Bundeswehrkrankenhäuser, die einer anderen Finanzierung unterliegen. Berufsgenossenschaftliche Kliniken sind medizinisch stark spezialisiert und entsprechen dem Grundgedanken einer Fachklinik. Bundeswehrkrankenhäuser entsprechen Allgemeinkrankenhäusern, die zahlreiche Leistungsbereiche vorhalten und über eine besondere Expertise bei Großschadensereignissen verfügen.

Bei der weiteren Ausarbeitung ist zu prüfen, ob weitere Besonderheiten bzw. eine Erweiterung der aufgeführten Definitionen erforderlich sind. Dazu ist eine empirische Evaluation hilfreich.

## 2 Welche Qualitätsmerkmale können für Fachkliniken beschrieben werden?

- Fachkliniken verfügen über überdurchschnittliche personelle und infrastrukturelle Merkmale, u.a. eine apparative Ausstattung, die auch die Betreuung seltener und komplexer Krankheitssituationen ermöglicht.
- Sie verfügen über eine fachspezifische Leitung durch mehrere Fachärzte und Fachärztinnen des Fachgebietes.
- Fachkliniken erfüllen Qualitätskriterien, die durch fachspezifische Zertifizierungen oder unabhängige Überprüfungen nachgewiesen werden.

## 3 Welchen Leveln können Fachkliniken zugeordnet werden?

Auch Fachkliniken sollen Leveln zugeordnet werden. Die Kriterien sind jedoch anzupassen aufgrund des heterogenen Behandlungsspektrums. Es wird deshalb vorgeschlagen, dass Fachkliniken einerseits anhand der Vorhaltung von Leistungsgruppen oder dem Umfang der abgedeckten ICD oder des Umfangs der OPs als auch andererseits anhand der vorgehaltenen Möglichkeiten der Notfall- und Intensivversorgung eingeordnet werden. Diese Optionen wurden gewählt, da das Kriterium der LG nur zielführend verwendet werden kann, wenn für das Fachgebiet mehrere (>2) LG festgelegt wurden. 

Im Folgenden sind die unterschiedlichen Möglichkeiten aufgeführt, inklusive der Berücksichtigung von hochspezialisierten Fachkliniken (Fachklinik S), für die eine gesonderte Level-Kennzeichnung ausgewiesen wird.

- **Level II S:** Fachklinik S, die die folgenden Kriterien erfüllt:
  - Auf die Diagnostik und Therapie einzelner Erkrankungen oder die Durchführung einzelner Behandlungen spezialisiertes Krankenhaus, das nicht die Kriterien von Level II A oder III erfüllt.
- **Level II A: **Fachklinik A, die jeweils ein Kriterium aus den Gruppen A und B erfüllt:
  - A: >1 LG des Fachgebietes ODER mindestens 60 bis <80% der ICD des Fachgebietes
  - B: Notfallversorgung 24/7 und Intensivstation (mindestens 4 Betten Beatmung) ODER 60 bis <80% der OPS des Fachgebietes
- **Level III:** Fachklinik A, die jeweils ein Kriterium aus den Gruppen A und B erfüllt:
  - A: Alle LG des Fachgebietes ODER ≥80% der ICD des Fachgebietes inklusive komplexer und seltener Diagnosen
  - B: Notfallversorgung 24/7 und Intensivstation mit Organersatzverfahren (mindestens 12 Betten Beatmung) ODER ≥80% der OPS des Fachgebietes inklusive (hoch-)komplexer Prozeduren
- **Level III (U):** Fachklinik des Level III, die durch Anbindung an eine Universität für diese wesentliche Teile von Lehre im Fachgebiet übernehmen (nicht nur Ausbildung im praktischen Jahr) und eine regelmäßige wissenschaftliche Tätigkeit nachweisen

## Anmerkung

Die folgenden Definitionsvorschläge für Fachkliniken wurden im April 2023 von den Autor*innen als Mitglieder der Ad-hoc-Kommission Versorgungsstrukturen der AWMF erarbeitet. Die Vorschläge werden von der gesamten Ad-hoc-Kommission Versorgungsstrukturen unterstützt.

## Interessenkonflikt

Die Autor*innen erklären, dass sie keine Interessenkonflikte in Zusammenhang mit diesem Artikel haben.

## References

[1] Regierungskommission für eine moderne und bedarfsgerechte Krankenhausversorgung (2022). Dritte Stellungnahme und Empfehlung der Regierungskommission für eine moderne und bedarfsgerechte Krankenhausversorgung. Grundlegende Reform der Krankenhausvergütung.

[2] Bundesministerium für Gesundheit (2023). Ergebnispapier 4. Sitzung der Facharbeitsgruppe zur Umsetzung der Krankenhausreform - Krankenhaus-Versorgungsstufen (Level).

[3] Ministerium für Arbeit, Gesundheit und Soziales des Landes Nordrhein-Westfalen (2022). Krankenhausplan Nordrhein-Westfalen 2022. Die Strukturen müssen für die Menschen da sein, nicht die Menschen für die Strukturen!.

[4] Bayerisches Staatsministerium für Gesundheit und Pflege (2023). Krankenhausplan des Freistaates Bayern.

[5] Sozialgesetzbuch (SGB) Fünftes Buch (V) - Gesetzliche Krankenversicherung - (Artikel 1 des Gesetzes v. 20. Dezember 1988, BGBl. I S. 2477) § 108 Zugelassene Krankenhäuser.

